# Finding Satisfaction With Life Through Serious Leisure: Older Men and Railroad Modeling

**DOI:** 10.1093/geroni/igaf122.3066

**Published:** 2025-12-31

**Authors:** Melinda Heinz, Hillary Wehe

**Affiliations:** University of Northern Iowa, Cedar Falls, Iowa, United States; University of Northern Iowa, Cedar Falls, Iowa, United States

## Abstract

This study aimed to provide support for railroad modeling as a serious leisure activity for older adult males and explore its relationship with their satisfaction with life. A convenience sample of males (n = 31) attending a regional railroad modeling conference were recruited. Participants completed the serious leisure inventory measure (SLIM) and satisfaction with life scale (SWLS). Hierarchical regression showed that serious engagement in railroad modeling positively predicted increases in life satisfaction, controlling for the physical health of the participants (R2 = .51, adjusted R2 = 0.47, F(2, 28)= 14.0, p <.001). A subsample of participants also completed interviews (n = 9) which were analyzed using Braun and Clarke’s reflexive thematic analysis. Theme 1: Adaptability of the Hobby, demonstrated diverse ways men could be involved and benefit from the hobby. Some were drawn to creative aspects while others were focused on socially connecting to other modelers. . Conferences and special interest groups provided opportunities to learn new skills. Theme 2: Lifelong Identity as a Railroad Modeler, reflected its consistent presence in the lives of most men, allowing them to take comfort in turning to the hobby during times of transition or stress. These findings highlight benefits of serious leisure involvement, particularly engagement in outlets that are adaptive . This allows men to participate in different ways with varied ability levels. This suggests that despite health changes, men may be able to remain involved with the hobby by modifying their participation while still maintaining their identity as a railroad modeler.

